# Integrative multi-omics analysis unravels the metabolic landscape and reveals serum biomarkers for early diagnosis of hyperuricemia

**DOI:** 10.1007/s11306-026-02509-2

**Published:** 2026-07-23

**Authors:** Xin Sun, Baoying Gong, Ye Sun, Hui Sun, Bin Zhang, Le Yang, Wenkai Wang, Qubo Chen, Shuyun Wei, Hao Wen, Ruicheng Liu, Ling Kong, Ying Han, Jianwen Guo, Xijun Wang

**Affiliations:** 1 State Key Laboratory of Dampness Syndrome of Chinese Medicine, The Second Affiliated Hospital of Guangzhou University of Chinese Medicine, Dade Road 111, Guangzhou, China; 2 State Key laboratory of Integration and Innovation for Classic Formula and Modern Chinese Medicine, National Chinmedomics Research Center, Metabolomics Laboratory, Department of Pharmaceutical Analysis, Heilongjiang University of Chinese Medicine, Heping Road 24, Harbin, 150040 China; 3 Department of Neurology, Guangdong Provincial Hospital of Chinese Medicine, Guangzhou, China; 4 Guangzhou Higher Education Mega Center, Guangzhou University of Chinese Medicine, No. 232, Waihuan East Road, Panyu District, Guangzhou, 510006 Guangdong Province China

**Keywords:** Hyperuricemia, Metabolomics, Proteomics, Multi-omics, Machine learning, Diagnostic biomarker

## Abstract

**Background:**

Hyperuricemia (HUA) is a major risk factor for gout and multiple metabolic disorders. Although serum uric acid (UA) is the gold standard for HUA diagnosis, it fails to reflect early metabolic disturbances and shows limited predictive value for asymptomatic HUA. This study sought to elucidate the pathological mechanisms underlying HUA and identify novel diagnostic biomarkers beyond UA.

**Methods:**

This study enrolled 195 patients with HUA and 98 healthy controls. Global metabolomics and proteomics profiling were performed to characterize molecular alterations underlying HUA. Based on the biological relevance of the shared dysregulated pathways, a pathway correlation network was constructed to elucidate the pathological mechanisms driving HUA initiation and progression. Furthermore, diagnostic biomarkers for HUA were identified using machine learning algorithms, and were validated with an external cohort.

**Results:**

HUA patients exhibited distinct metabolic and proteomic profiles compared with healthy controls. Integrated multi-omics pathway analysis revealed that peroxisome proliferators-activated receptor signaling pathway, arachidonic acid metabolism, purine metabolism, pyrimidine metabolism and sphingolipid signaling pathway were significantly dysregulated in HUA. Among them, arachidonic acid metabolism was identified as a hub pathway involved in HUA progression. Furthermore, a metabolite panel consisting of cysteine-S-sulfate, glycerophosphocholine and 4-hydroxyphenylpyruvic acid was screened by machine learning and validated in an independent cohort, which showed slightly higher diagnostic performance for HUA than UA.

**Conclusions:**

This study reveals the core metabolic and protein regulatory networks of HUA, and identifies a novel serum metabolite panel for the diagnosis of HUA. These findings provide new insights for improved clinical diagnosis and management.

**Supplementary Information:**

The online version contains supplementary material available at https://doi.org/10.1007/s11306-026-02509-2.

## Introduction

Hyperuricemia (HUA) is a prevalent metabolic disorder resulting from an imbalance in purine metabolism and kidney excretion, characterized by the excessive production of uric acid (UA) (Dalbeth et al., 2021). The prevalence of HUA is on the rise, especially in Asian countries such as China, where it has reached 18.4% (Gao et al., 2022). HUA has become the fourth most important “hyper condition” after hypertension, hyperlipidemia, and hyperglycemia (Guo et al., 2020). HUA is linked to various pathological conditions, including metabolic syndrome, cardiovascular diseases, and renal diseases (Dehlin et al., 2020). Besides, the abnormal elevation of serum UA may cause inflammation and vasoconstriction in patients with HUA. The clinical diagnostic criteria for clinically assessing and diagnosing HUA involve imaging techniques and the analysis of urine, blood, and renal function indicators (Yip et al., 2020). In the asymptomatic phase, the onset of HUA often goes undetected (Richette et al., 2020). It is concerning that many asymptomatic HUA patients without gout exhibited multiple cardiovascular risk factors or have kidney disease and diabetes (Johnson et al., 2018). The lack of consensus among clinicians on treating asymptomatic HUA often leads to delayed diagnosis in early stages. Therefore, there is a pressing demand for innovative predictive biomarkers to allow for the early detection of HUA.

Metabolomics employs high-throughput techniques to identify, quantify and characterize small-molecule metabolites, providing a powerful tool for discovering disease-related metabolic biomarkers. By combining untargeted and targeted metabolomic strategies, Qin et al. screened and identified nine differential metabolites between HUA patients and controls, including L-tyrosine, L-phenylalanine, arachidonic acid, linoleic acid, oleic acid, stearic acid, LysoPC(18:0), LysoPC(18:1(9Z), and LysoPC(16:0) (Qin et al., 2022). Another serum metabolomics study revealed that metabolic syndrome combined with HUA induces profound perturbations in lipid, amino acid, glucose-energy metabolism and redox homeostasis. Several metabolites, including glutamate, glutamine, citrate, lysine, very low-density lipoprotein, triglycerides, and trimethylamine N-oxide, were recognized as candidate metabolic biomarkers. Collectively, these findings indicate that elevated UA aggravates metabolic abnormalities, and proper UA regulation may slow the progression of metabolic syndrome (Zhang et al., 2021). The Q-Exactive mass spectrometer, with its high sensitivity and rapid polarity switching capabilities, is widely validated for metabolomics applications (Li et al., 2023). These features make it particularly well-suited for the untargeted metabolomics analysis in this study, enabling comprehensive profiling of metabolic perturbations underlying HUA and maximizing coverage of pathways relevant to its pathophysiology.

As a downstream functional layer of the genome and transcriptome, the proteome is more closely linked to ultimate metabolic phenotypes (Geyer et al., 2017). It facilitates in-depth exploration of cellular pathways and regulatory mechanisms, and has been widely employed for biomarker discovery and novel therapeutic target identification. Emerging evidence has demonstrated that patients with HUA display significant dysregulation of multiple proteins relative to controls, including V-type proton ATPase subunit B, complement factor D, and apolipoprotein C 3 (Huo et al., 2021).

Nevertheless, the specific diagnostic biomarkers and precise molecular mechanisms underlying HUA remain to be fully elucidated. Relying exclusively on proteomics analysis alone to characterize key processes in HUA may bias results toward highly abundant proteins, rather than those most functionally relevant to the disease (Huo et al., 2021). By contrast, excessive reliance on metabolomics analyses may lead to ambiguous interpretations of core biological processes (Cui et al., 2017). Fortunately, clinical multi-omics approaches offer a novel framework for integrating pathological phenotypes, thereby enabling an in-depth understanding of disease pathogenesis from multiple dimensions (Jin et al., 2021; Wigger et al., 2021). This approach facilitates exploration of complex global regulatory networks (Xu et al., 2019). The combined application of proteomics and metabolomics has been widely adopted in studies of cardiomyopathy (Li et al., 2020), inflammatory bowel disease (Lee et al., 2021), non-small cell lung cancer (Xu et al., 2023), and hepatitis C infection (Ali et al., 2023).

Accordingly, the present study integrates proteomics and metabolomics approaches to fill the existing gap in the systematic mechanistic investigation of HUA. We first performed proteomics and metabolomics profiling to compare HUA patients with healthy control (HC), aiming to comprehensively characterize the molecular perturbations and pathological mechanisms underlying HUA. Second, we screened and identified potential diagnostic biomarkers for HUA by integrating metabolomics with machine learning algorithms, followed by validation in an independent cohort. Collectively, this multi-omics approach delineates a holistic landscape of molecular alterations in HUA, which may yield novel insights into its clinical diagnosis, pathogenesis, and prognostic evaluation.

## Methods

### Chemicals and reagents

All chemicals, reagents, and solvents are detailed in the Supporting Information.

### Collections and preparation of clinical specimen

A total of 195 HUA patients and 98 HCs from Guangzhou No. 11 People’s Hospital were enrolled in this study between June and July 2022. The discovery cohort consisted of 81 HUA patients and 42 HCs, while the validation cohort included 114 HUA patients and 56 HCs. Inclusion criteria included male participants aged over 18 years. All HUA patients had fasting serum UA levels greater than 420 µmol/L on two separate occasions and had no current or prior gout symptoms. In accordance with the 2015 EULAR/ACR classification criteria for gout (Neogi et al., 2015), none of the enrolled patients presented with swelling, pain, or tenderness in peripheral joints or bursae, and monosodium urate crystals were not detected in any subject. Individuals with severe cardiovascular, cerebrovascular, pulmonary, hepatic, renal diseases, hematological malignancies, or psychiatric disorders were excluded. HCs were age-matched to the patient group, with no self-reported chronic illnesses and normal physical examination findings.

Anthropometric and biochemical data were collected from all participants, including age, body mass index (BMI), fasting blood glucose, serum UA, and other relevant indicators. Following an overnight fast and off all medications, all participants donated fasting venous blood samples for subsequent laboratory analysis.

Whole blood samples were collected from all participants and allowed to clot at room temperature for 30 min. Subsequently, the samples were centrifuged at 4000 rpm for 15 min at 4 °C to isolate serum. The separated serum was stored at − 80 °C until further analysis.

### Metabolomics analysis

Detailed protocols for serum metabolomics analysis, including sample preparation, UPLC-MS measurement and subsequent data analysis, are provided in the Supplementary Information.

### Proteomics analysis

Detailed protocols for serum proteomics analysis, including sample preparation, UPLC-MS measurement and subsequent data analysis, are provided in the Supplementary Information.

### Bioinformatics analysis

#### Joint analysis of metabolomics and proteomics

KEGG pathway enrichment analysis was performed for both differential metabolites and differential proteins to screen overlapping enriched pathways. Based on the enrichment results, we further explored the intrinsic interrelationships among these enriched proteins and metabolites underlying HUA pathogenesis. All molecules were systematically annotated to corresponding pathways to elucidate the potential molecular mechanisms involved in HUA progression. The interaction network was visualized using Cytoscape (Shannon et al., 2003), and the KGML database from KEGG was adopted to characterize the crosstalk among proteins and metabolites.

#### Development of a diagnostic model of HUA

To enhance diagnostic model accuracy, a metabolite-based model was developed using support vector machine (SVM), partial least squares discriminant analysis (PLS-DA), and random forest (RF). Receiver operating characteristic (ROC) curves were generated using 10-fold cross-validation. The analysis was conducted using the R package MetaboAnalyst R. Area under the curve (AUC) was implemented to evaluate the diagnostic performance of potential biomarkers with classification cutoff-specific evaluation metrics, such as sensitivity and specificity.

### Statistical analysis

Statistical analysis was performed using GraphPad Prism (v8.01, San Diego, CA, USA). Spearman correlation was used for the analysis. Statistical significance was determined by a *P* < 0.05.

## Results

### Baseline characteristics of the participants

Clinical and biochemical characteristics were compared between HUA patients and HCs in both the discovery and validation cohorts. Baseline demographic and clinical profiles of the discovery and validation cohort are summarized in Table 1. Age and fasting blood glucose levels were comparable between the two groups (*P* > 0.05). Compared with the HC group, the HUA group had significantly higher BMI, systolic blood pressure (SBP), diastolic blood pressure (DBP), alanine transaminase (ALT), aspartate transaminase (AST), serum creatinine, serum UA, total cholesterol (TC), triglyceride (TG), and low-density lipoprotein cholesterol (LDL-C) (*P* < 0.05). The external validation cohort exhibited similar baseline demographic and biochemical characteristics to the discovery cohort. Collectively, these findings indicated that HUA patients were prone to concomitant obesity and hyperlipidemia, and such metabolic abnormalities were closely correlated with systemic metabolic disorders.

**Table 1:** Baseline characteristics of the participants in two cohorts

Variables	HC in the discovery cohort (*N* = 42)	HUA in the discovery cohort (*N* = 81)	HC in the validation cohort (*N* = 56)	HUA in the validation cohort (*N* = 114)
Age (years)	44.86 ± 9.04	44.36 ± 9.6	45.29 ± 10.2	44.42 ± 9.16
BMI (Kg/m^2^)	23.58 ± 1.95	25.35 ± 2.92**	24.03 ± 2.64	24.87 ± 2.74
SBP (mmHg)	115.07 ± 11.98	120.08 ± 11.19*	118.25 ± 12.07	120.78 ± 14.97
DBP (mmHg)	69.31 ± 8.16	74.75 ± 8.52**	71.30 ± 8.02	73.04 ± 10.71
ALT (U/L)	17.33 ± 5.83	22.07 ± 7.23***	20.29 ± 8.01	21.47 ± 7.3
AST (U/L)	17.40 ± 3.07	18.9 ± 4.24*	18.66 ± 4.33	19.07 ± 3.37
Creatinine (µmol/L)	84.05 ± 9.99	89.64 ± 9.13**	83.1 ± 11.87	88.54 ± 10.39**
UA (µmol/L)	358.47 ± 48.35	491.73 ± 57.65***	351.83 ± 60.15	484.13 ± 58.85***
Fasting glucose (mmol/L)	5.23 ± 0.60	5.22 ± 0.41	5.4 ± 0.94	5.16 ± 0.48*
TC (mmol/L)	4.20 ± 0.62	4.95 ± 0.8***	4.43 ± 0.57	4.84 ± 0.80**
TG (mmol/L)	1.04 ± 0.32	1.59 ± 1.16**	1.11 ± 0.27	1.77 ± 1.16***
HDL-C (mmol/L)	1.39 ± 0.32	1.37 ± 0.29	1.37 ± 0.26	1.27 ± 0.24**
LDL-C(mmol/L)	2.53 ± 0.60	3.16 ± 0.72***	2.76 ± 0.52	3.09 ± 0.77**

Data are expressed as mean ± standard deviation. Comparison between HC and HUA: *P<0.05, **P<0.01, ***P<0.001

### Metabolites such as arachidonic acid, linoleic acid and sphingosine are associated with HUA in discovery cohort

**Fig. 1:**
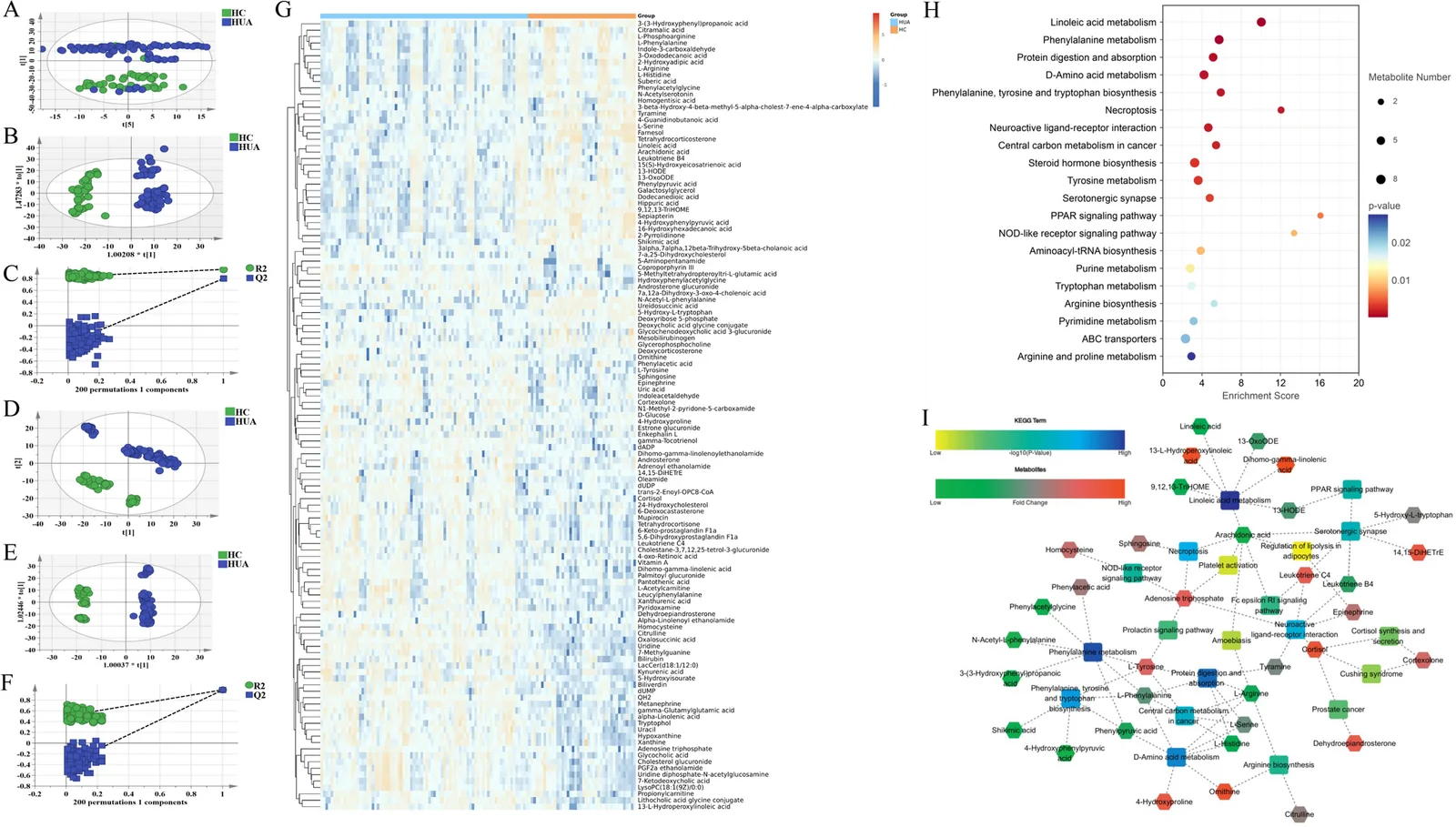
Screening and enrichment analysis of differential metabolite in discovery cohort. **A**–**C** PCA, OPLS-DA scatter plots, and permutation test plots in positive ions. **D**–**F** PCA, OPLS-DA scatter plots, and permutation test plots in negative ions. **G** Heatmap of the abundance profiles of differential metabolites between the HUA and HC groups. **H** Bubble plot of KEGG pathway enrichment analysis for differential metabolites. **I** Network diagram of metabolite–pathway interactions. Hexagons denote metabolites, and squares represent KEGG pathways

After UPLC/MS detection and data preprocessing, 7395 and 4278 metabolic features were identified in the discovery cohort under positive and negative ion modes, respectively. PCA revealed clear intergroup separation in both modes (Fig. 1A and D). Supervised OPLS-DA further enhanced group discrimination (Fig. 1B and E), showing satisfactory model fitness between HUA and HC groups: R2X = 0.343, R2Y = 0.956, Q2 = 0.799 (positive mode); R2X = 0.277, R2Y = 0.99, Q2 = 0.985 (negative mode), confirming distinct serum metabolomics signatures. Two hundred permutation tests validated no overfitting of the OPLS-DA models (Fig. 1C and F), with all permuted R2 and Q2 values lower than original ones and a negative intercept of the Q2 regression line. Differential metabolites were defined as *P* < 0.05 and FC > 1.2 or < 0.83; annotation via HMDB database fragment matching identified 123 differential metabolites in the HUA group, including arachidonic acid, linoleic acid, and sphingosine (Fig. 1G).

KEGG pathway enrichment analysis was performed to identify meaningful metabolic patterns and perturbed pathways (Table S2). The top 20 statistically significant pathways of differential metabolites are shown in Fig. 1H, including linoleic acid metabolism, phenylalanine metabolism, protein digestion and absorption, D-amino acid metabolism, and phenylalanine, tyrosine and tryptophan biosynthesis. A network interaction diagram (Fig. 1I) visualized key metabolites and their associated pathways, highlighting the central role of linoleic acid metabolism and phenylalanine metabolism; critical metabolites (linoleic acid, arachidonic acid, phenylalanine) act as key nodes linking multiple biological processes, including peroxisome proliferators-activated receptor (PPAR) signaling pathway, neuroactive ligand-receptor interaction, and platelet activation.

### Proteins such as RHOA, KRAS and AGT are associated with HUA

**Fig. 2:**
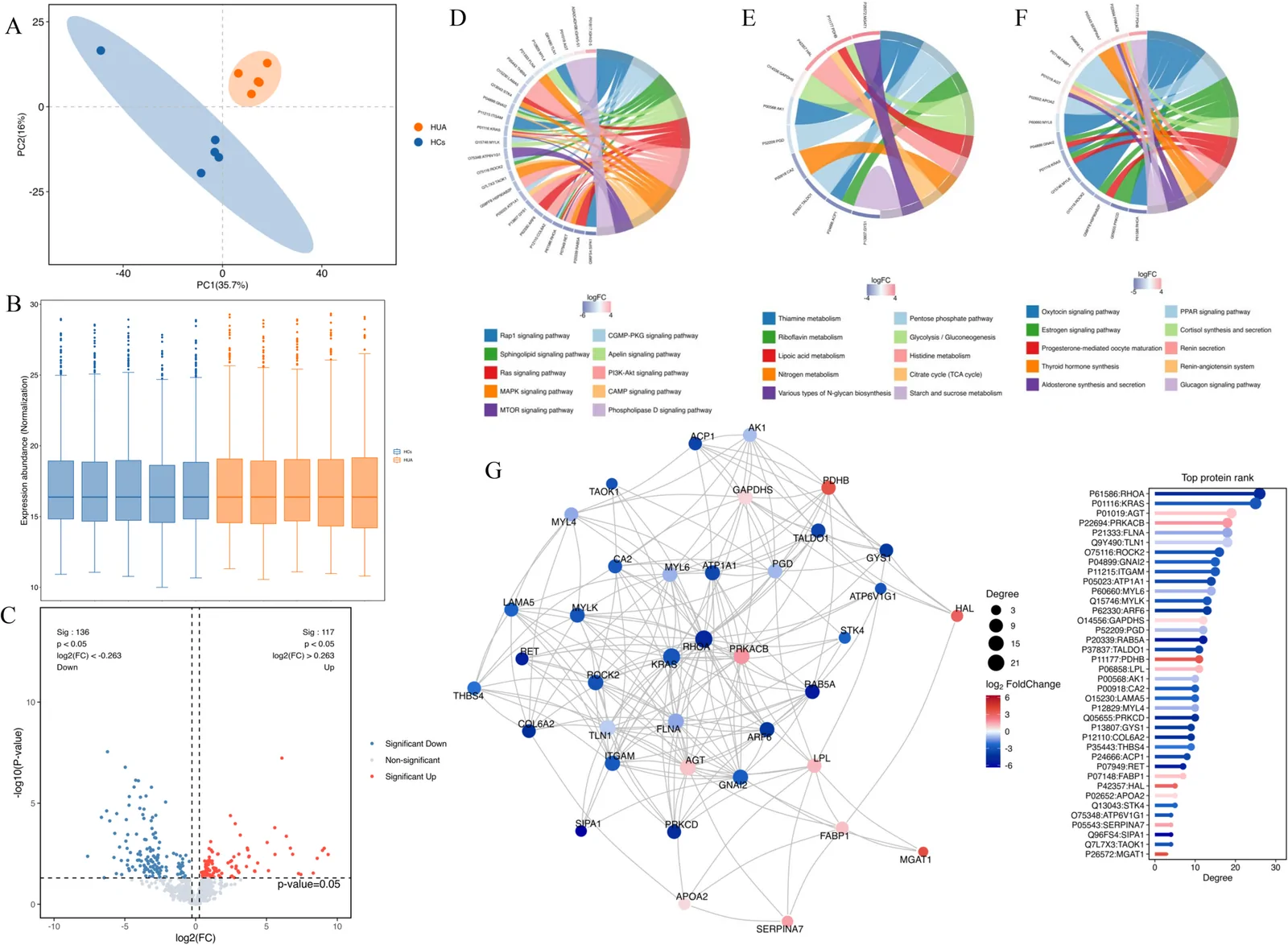
Screening and enrichment analysis of differential protein. **A** PCA scatter plot showed that the protein data of HCs and HUA patients had good separation characteristics. **B** Boxplots of normalized protein expression abundance in HC and HUA groups. **C** Volcano map of 253 differential protein screening. **D**–**F** Chord diagram of the differentially abundant proteins to KEGG pathways of environmental information processing, metabolism and organismal systems, respectively. **G** PPI network of differentially expressed proteins

To identify key proteins and molecular pathways associated with HUA pathogenesis, we performed comprehensive proteomic analysis on the discovery cohort. Rigorous quality control confirmed the MS platform’s stability and repeatability (Fig. S1), and clustering tree analysis determined the optimal pre-processing method (Fig. S2). After data processing, 1066 protein features were detected (Fig. S3). PCA showed clear separation between HUA and HC groups, indicating distinct proteomic profiles (Fig. 2A). Boxplots of normalized protein expression abundance exhibited consistent, comparable distributions between groups, supporting data reliability (Fig. 2B). Using *P* < 0.05 and FC > 1.2 or < 0.83, 253 significantly altered proteins were identified in HUA patients, including 117 upregulated and 136 downregulated proteins (Fig. 2C).

KEGG pathway enrichment analysis was conducted to clarify the functions of differentially expressed proteins (Table S3). Visualization via chord diagrams revealed significant enrichment in three primary categories: environmental information processing (Fig. 2D), metabolism (Fig. 2E), and organismal systems (Fig. 2F). Key pathways included signaling cascades, amino acid/carbohydrate metabolism, and endocrine/immune regulation, indicating broad perturbations in signal transduction, metabolic homeostasis, and systemic regulation in HUA. PPI network analysis identified key hub proteins (Fig. 2G); the highly interconnected network’s top hub proteins (ranked by degree centrality) were RHOC, AGT, TLN1, and PLG, which are involved in cytoskeletal regulation, signal transduction, and metabolic enzyme activity, suggesting their potential central roles in HUA pathogenesis.

### Interaction between proteomics and metabolomics suggests arachidonic acid metabolism as a mediator in HUA

**Fig. 3:**
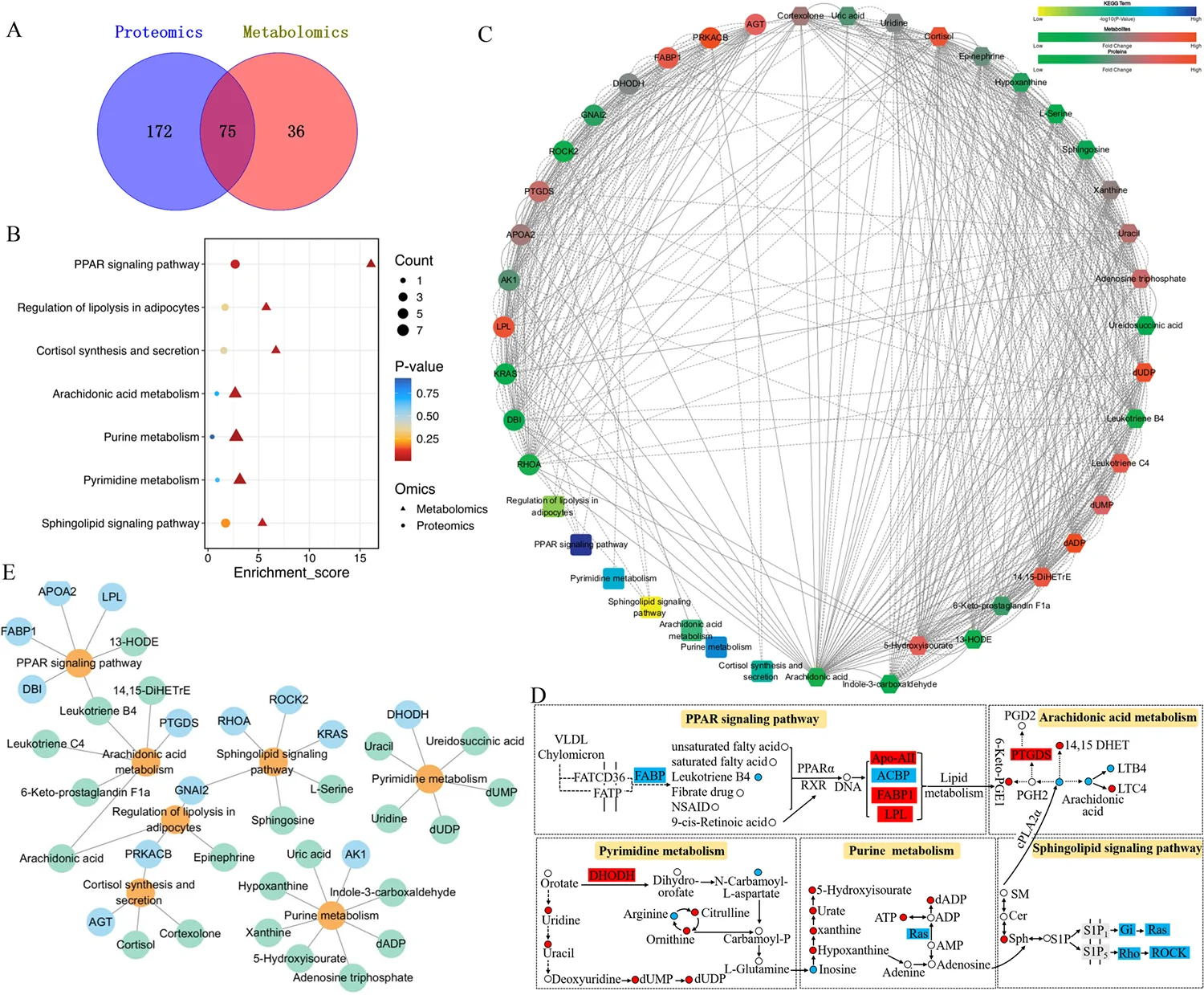
Integration landscape of metabolomics and proteomics in HUA. **A** Venn plot of 75 pathways common to metabolomics and proteomics. **B** Bubble plot of 7 significant changes common paths associated with HUA. **C** Integrated interaction network of differentially expressed metabolites and proteins. **D** Detailed map of metabolite–protein–pathway associations. **E** Crosstalk network of key metabolic pathways involved in HUA

To screen HUA-associated key metabolites/proteins and their core biological pathways, we performed multi-omics integration of metabolomic and proteomic profiles. A total of 75 pathways were commonly enriched (Fig. 3A), with the most significant overlapping ones (*P* < 0.05) identified in Fig. 3B and Table S4, including PPAR signaling pathway, lipolysis regulation in adipocytes, cortisol synthesis and secretion, arachidonic acid metabolism, purine/pyrimidine metabolism, and sphingolipid signaling pathway. An integrated interaction network (Fig. 3C) dissected metabolite-protein crosstalk, showing a highly interconnected topology centered on PPAR signaling, arachidonic acid metabolism, and purine metabolism. Detailed pathway maps demonstrated that dysregulated proteins and metabolites coordinately disrupted lipid homeostasis and inflammatory responses (Fig. 3D). A pathway crosstalk network (Fig. 3E) characterized associations among core pathways, differential proteins, and metabolites: key interactions included PPAR signaling linked to APOA2, LPL, FABP1, DBI and 13-HODE, and arachidonic acid metabolism connected to PTGDS, RHOA, GNAI2 and metabolites (leukotriene B4, 14,15-DiHETrE, leukotriene C4, 6-keto-prostaglandin F1a, arachidonic acid).

### The combination of cysteine-S-sulfate, glycerophosphocholine and 4-hydroxyphenylpyruvic acid enables more accurate diagnosis of HUA than UA

To develop and validate a precise HUA diagnostic model, we performed metabolomics analysis of the validation cohort and machine learning-based screening in both discovery and validation cohorts. PCA showed a grouping tendency in the validation cohort; OPLS-DA models separated HUA and HC samples with satisfactory fitness (positive mode: R2X = 0.348, R2Y = 0.943, Q2 = 0.858; negative mode: R2X = 0.233, R2Y = 0.984, Q2 = 0.980) and were validated as valid (Fig. S4). Candidate differential biomarkers were screened by *P* < 0.05 and FC > 1.2 or < 0.83, further filtered by AUC > 0.6 in both cohorts, and annotated via HMDB and KEGG databases. Finally, 26 reliable metabolic signatures (including cysteine-S-sulfate, glycerophosphocholine, hippuric acid) were consistently identified across the two cohorts (Table S5).

**Fig. 4:**
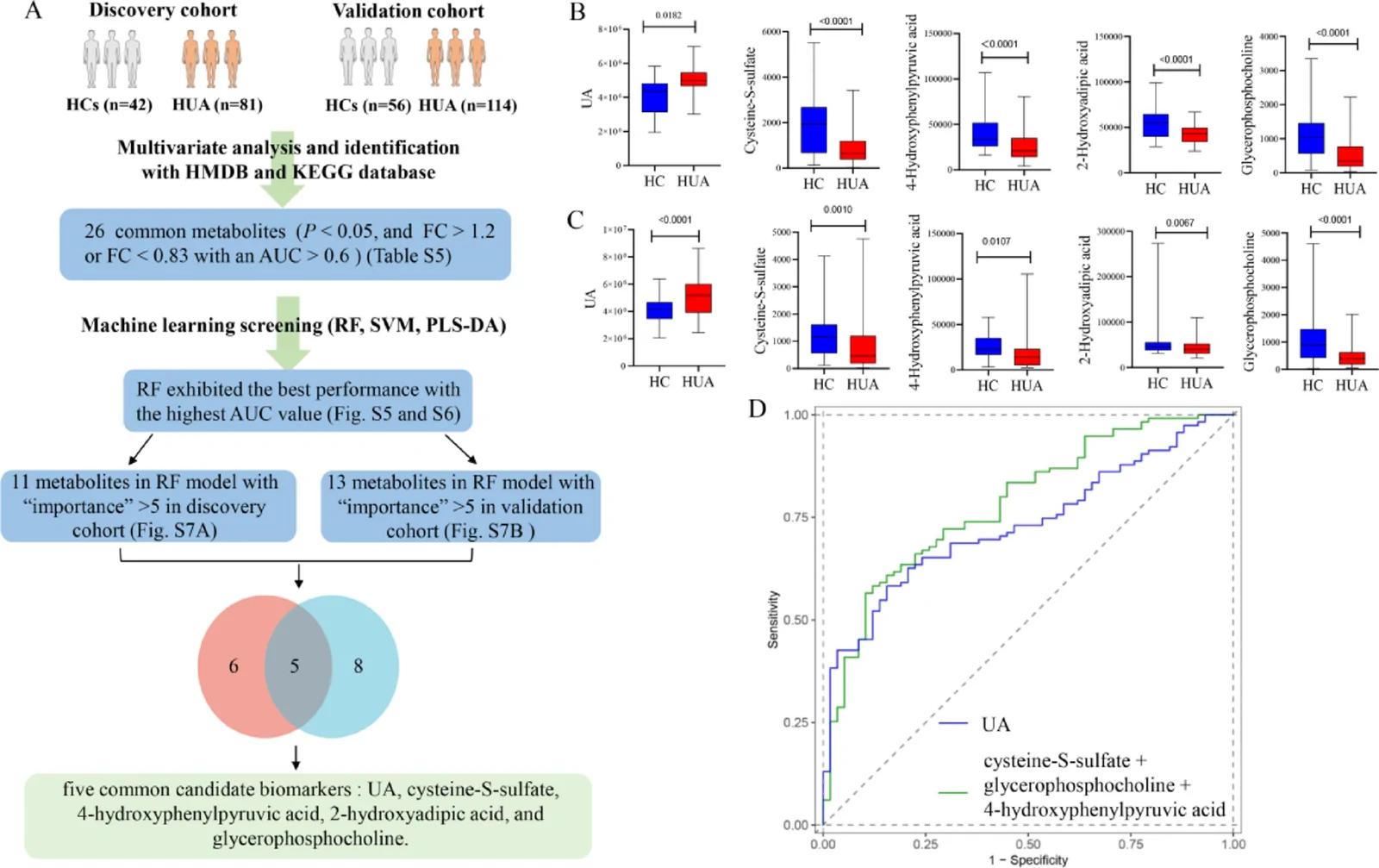
Diagnostic model development and validation for HUA. **A** Flowchart of methodology for screening diagnostic biomarkers. **B**–**C** The expression levels of three diagnostic metabolites of HUA in discover and validation cohorts. **D** ROC curves demonstrating the optimal metabolite panel outperformed UA alone for distinguishing HUA patients from HC

To establish an optimal HUA diagnostic predictive model, we applied three machine learning algorithms (SVM, RF, PLS-DA). In the discovery cohort, RF achieved the highest AUC (0.969; 95% CI: 0.890–1), outperforming SVM (0.950; 95% CI: 0.883–1) and PLS-DA (0.895; 95% CI: 0.847–0.945) (Fig. S5A–C), with probability distribution plots showing the clearest HUA-HC separation (Fig. S5D–F). This superiority was replicated in the validation cohort: RF maintained the highest AUC (0.931; 95% CI: 0.864–0.964), exceeding SVM (0.884; 95% CI: 0.792–0.962) and PLS-DA (0.824; 95% CI: 0.734–0.894) (Fig. S6A–C) and the most distinct group separation (Fig. S6D–F), supporting RF as the optimal model for subsequent biomarker screening. RF “importance” (quantifying metabolite contribution to reducing node impurity) with an “average importance > 5” threshold selected 11 metabolites from the discovery cohort (Fig. S7A) and 13 from the validation cohort (Fig. S7B). Following the workflow (Fig. 4A), five common metabolites were identified: UA, cysteine-S-sulfate, 4-hydroxyphenylpyruvic acid, 2-hydroxyadipic acid, and glycerophosphocholine, whose expression profiles were validated in both cohorts (Fig. 4B and C). Diagnostic performance of 4-hydroxyphenylpyruvic acid, glycerophosphocholine, 2-hydroxyadipic acid, cysteine-S-sulfate, and their combinations was evaluated in the validation cohort (Table 2, Fig. S8). Finally, a three-metabolite panel (cysteine-S-sulfate, glycerophosphocholine, 4-hydroxyphenylpyruvic acid) was identified, showing robust performance and superior efficacy to UA alone in distinguishing HUA from HC (Fig. 4D).

**Table 2:** Performance of 5 potential biomarkers and different combinations of permutations in the validation cohort between HUA patients and HC

Feature	AUC	Specificity	Sensitivity	cutoff	95%CI
UA	0.733	0.845	0.583	5,008,234	0.659–0.807
cysteine-S-sulfate	0.702	0.707	0.670	772	0.624–0.781
4-hydroxyphenylpyruvic acid	0.706	0.828	0.574	16,146	0.628–0.783
2-hydroxyadipic acid	0.659	0.948	0.426	34,804	0.579–0.739
glycerophosphocholine	0.702	0.586	0.791	629	0.615–0.790
cysteine-S-sulfate + 4-hydroxyphenylpyruvic acid	0.715	0.862	0.504	0.753	0.639–0.792
cysteine-S-sulfate + 2-hydroxyadipic acid	0.743	0.759	0.730	0.684	0.669–0.817
glycerophosphocholine + 2-hydroxyadipic acid	0.745	0.603	0.783	0.651	0.668–0.822
2-hydroxyadipic acid + 4-hydroxyphenylpyruvic acid	0.688	0.966	0.400	0.747	0.611–0.765
glycerophosphocholine + 4-hydroxyphenylpyruvic acid	0.753	0.845	0.574	0.760	0.678–0.829
cysteine-S-sulfate + glycerophosphocholine	0.740	0.690	0.704	0.706	0.659–0.821
cysteine-S-sulfate + glycerophosphocholine + 4-hydroxyphenylpyruvic acid	0.784	0.879	0.583	0.769	0.714–0.854
glycerophosphocholine + 2-hydroxyadipic acid + 4-hydroxyphenylpyruvic acid	0.763	0.862	0.548	0.775	0.689–0.836
cysteine-S-sulfate + glycerophosphocholine + 2-hydroxyadipic acid	0.769	0.707	0.739	0.692	0.695–0.843
cysteine-S-sulfate + glycerophosphocholine + 2-hydroxyadipic acid + 4-hydroxyphenylpyruvic acid	0.775	0.810	0.635	0.740	0.703–0.848

Cutoff values for single metabolites correspond to raw MS signal intensities; cutoff values for combined metabolite panels represent model-predicted positive risk probabilities

## Discussion

In clinical practice, UA is the only biochemical indicator for HUA diagnosis, limiting its early prevention. Herein, integrated metabolomics and proteomics identified HUA’s molecular signatures and core regulatory networks, with arachidonic acid metabolism as a central hub in pathogenesis. Via machine learning and an independent external cohort, we identified and validated a novel serum metabolite panel (cysteine-S-sulfate, glycerophosphocholine, 4-hydroxyphenylpyruvic acid), which exhibits superior HUA diagnostic efficiency to UA and compensates for UA’s limitation in detecting early metabolic disorders and asymptomatic HUA.

We observed marked downregulation of three metabolites within the diagnostic biomarker panel for HUA. Cysteine-S-sulfate is catabolized to glutathione for ROS neutralization; glutathione reductase recycles this antioxidant to sustain cellular redox stability and counteract oxidative stress (Dawi et al., 2024; He et al., 2025). Consistent with this antioxidant function, additional research has reported diminished cysteine-S-sulfate levels in heart failure patients, implying this metabolite exerts a vital regulatory effect on oxidative stress homeostasis (Wegermann et al., 2023). Previous studies have indicated glycerophospholipid govern inflammation, oxidative stress, insulin resistance and hyperglycaemia-elicited vascular complications (Castro-Gómez et al., 2015; Dang et al., 2018). Consistently, we found reduced glycerophosphocholine levels in HUA patients. Within the secondary oxidative branch of tyrosine metabolism, phenylalanine is converted to 4-hydroxyphenylpyruvic acid via tyrosine intermediation. Catalyzed by 4-hydroxyphenylpyruvate dioxygenase, 4-hydroxyphenylpyruvic acid is oxidized to homogentisic acid and subsequently catabolized into coenzyme A and fumaric acid (Endo et al., 2003). The two products then fuel the TCA cycle, the core cascade sustaining cellular energy metabolism (Heylen et al., 2012). Lu et al. reported suppressed circulating 4-hydroxyphenylpyruvic acid levels in type 1 diabetes (Lu et al., 2013), implying downregulation of HPPA in the tyrosine pathway may disrupt energy metabolic homeostasis in patients with metabolic diseases including HUA.

Integrated multi-omics correlation analysis identified arachidonic acid metabolism as a pivotal pathway in HUA pathogenesis. Elevated pro-inflammatory prostaglandin D2 synthase alters HUA patients’ inflammatory mediator profiles. Arachidonic acid is converted to 5-hydroperoxyeicosatetraenoic acid, then to leukotriene A4, which undergoes two metabolic pathways: leukotriene A4 is catalyzed by leukotriene C4 synthase to form leukotriene C4, which plays a critical pro-inflammatory role (Rengachar et al., 2022). Leukotriene A4 is directly hydrolyzed by leukotriene A4 hydrolase to generate leukotriene B4, thereby connecting the PPAR signaling pathway with arachidonic acid metabolism and exerting a pivotal role in HUA pathogenesis. 14, 15-DHET acts as a crucial downstream metabolite of arachidonic acid via the CYP450 pathway (Chen et al., 2022), and is closely involved in human inflammatory responses (Valdes et al., 2018). Arachidonic acid and its derivatives have diverse biological functions, highlighting their potential as HUA therapeutic targets.

PPARs are nuclear hormone receptors that govern glucose and lipid homeostasis (Li et al., 2022). PPARα modulates intracellular glycolipid metabolism by regulating genes related to peroxisomal and mitochondrial fatty acid uptake. In this study, HUA patients exhibited elevated levels of Apo-AII, FABP1, and LPL. High Apo-AII induces insulin resistance and lipid imbalance, and is closely associated with metabolic disorders (Castellani et al., 2001; Clarke et al., 2023). FABP1 regulates lipid metabolism and correlates with obesity and non-alcoholic fatty liver disease (Hostetler et al., 2009; Thumser et al., 2014). As a key lipolytic enzyme, LPL facilitates triglyceride hydrolysis and fatty acid uptake, and its upregulation contributes to insulin resistance and obesity (Abbas et al., 2022; Young et al., 2019). Taken together, our results indicate that activated PPARα signaling disturbs glycolipid metabolism and ultimately increases HUA susceptibility.

Purine and pyrimidine metabolism produces UA as the final product, and excessive UA synthesis or insufficient excretion triggers HUA (Zheng et al., 2018). In this study, HUA patients exhibited disturbances in purine and pyrimidine metabolism. Elevated adenosine triphosphate suppressed the phosphorylation of uridine diphosphate, leading to accumulation of uridine diphosphate, uridine monophosphate and their deoxy derivatives. Such alterations accelerated pyrimidine catabolism and raised serum uridine and uracil levels (Dudzinska et al., 2010). These two metabolites are tightly involved in inflammatory responses (Li et al., 2011; Motta et al., 2018). Accordingly, abnormal nucleotide metabolism facilitates UA accumulation and sustains inflammatory status in HUA.

## Limitations

Our findings revealed significant metabolomic and proteomic alterations in HUA. Importantly, multi-omics integration identified key pathogenic biochemical pathways, providing novel insights into the disease’s complex molecular mechanisms. Additionally, combining discovery/validation cohorts and machine learning allowed us to screen promising novel metabolic biomarkers for HUA diagnosis. Despite these promising insights, this study still has the following limitations: all participants enrolled in the present cohort were male, future large-scale cohorts containing both male and female HUA patients are required to validate the universality, sensitivity, specificity and accuracy of our diagnostic model, and the application value of these biomarkers in early HUA diagnosis still needs further verification. Besides, two deuterated amino acid internal standards fail to fully compensate matrix effects and extraction losses for other non-amino metabolites, leading to minor quantitative bias. Nevertheless, this study was implemented with rigorous and standardized experimental arrangements. As an exploratory research, it systematically elucidated the overall omics alterations in HUA and screened valid candidate biomarkers through in-depth multi-cohort analysis.

## Conclusions

This is the first study to systematically elucidate HUA pathogenic mechanisms via combined proteomics and metabolomics. Disordered lipid and nucleotide metabolism closely related to inflammation and UA homeostasis imbalance act as core drivers of HUA progression. We further verified that the three-metabolite panel, including cysteine‑S‑sulfate, glycerophosphocholine and 4‑hydroxyphenylpyruvic acid, achieves marginally improved diagnostic efficacy for HUA than serum UA. These findings offer new mechanistic perspectives for HUA pathogenesis, facilitate targeted therapeutic strategy development, and provide reliable references for clinical diagnosis and management of HUA.

## Supplementary Information

Below is the link to the electronic supplementary material.

Supplementary material 1 (DOCX 1543.2 kb)

## Data Availability

The datasets used and/or analyzed during the current study may be found within the manuscript and in the associated supplementary files. All data used in this study are available from the corresponding author upon reasonable request.

## Abbreviations

ALT: Alanine transaminase
AST: Aspartate transaminase
AUC: Area under the curve
BMI: Body mass index
DBP: Diastolic blood pressure
DIA: Data-independent acquisition
FC: Fold change
FDR: False discovery rate
HC: Healthy control
HMDB: Human metabolome database
HUA: Hyperuricemia
KEGG: Kyoto encyclopedia of genes and genomes
LDL-C: Low-density lipoprotein cholesterol
PCA: Principal component analysis
PLS-DA: Partial least squares discriminant analysis
OPLS-DA: Orthogonal partial least squares discriminant analysis
PPI: Protein‑protein interaction
RF: Random forest
ROC: Receiver operating characteristic
SBP: Systolic blood pressure
SVM: Supporting vector machine
TC: Total cholesterol
TG: Triglyceride
UA: Uric acid
