# Supplementary material for: Integrative multi-omics analysis unravels the metabolic landscape and reveals serum biomarkers for early diagnosis of hyperuricemia
Source: Metabolomics. 2026 Jul 23;22(4):130. doi: 10.1007/s11306-026-02509-2 (PMC13396029; doi:10.1007/s11306-026-02509-2)
Supplement: Supplementary file 1 — Supplementary material 1 (DOCX 1543.2 kb) [file 11306_2026_2509_MOESM1_ESM.docx]

**Supplementary Information**

**Integrative multi-omics analysis unravels the metabolic landscape and reveals serum biomarkers for early diagnosis of hyperuricemia**

**Methods**

**Chemicals and reagents**

Dithiothreitol, iodoacetamide, ammonium bicarbonate and acetonitrile were purchased from MilliporeSigma. Trifluoroacetic acid, iRT calibration standards and formic acid were obtained from Thermo Fisher Scientific. Sequencing-grade modified trypsin was supplied by Thermo Fisher Pierce. Deuterated L-phenylalanine and deuterated L-tryptophan isotopic standards were provided by Andover. Methanol was supplied by Fisher Chemicals. All chemicals and solvents used were LC‑MS grade.

**Metabolomics analysis**

**Sample preparation**

Serum metabolites were extracted by adding 200 μL of cold methanol to 50 μL of serum, followed by 1 min of vortex mixing and 20 min of incubation at 4 °C, then centrifuged at 13000 rpm for 15 min under 4 °C. The supernatant was filtered through a 0.22 μm membrane for UPLC-MS analysis. A quality control (QC) sample was prepared by pooling all serum samples aliquots.

A mixture of deuterated L-phenylalanine and deuterated L-tryptophan was added to each serum sample as an internal standard for normalization of metabolite peak areas to correct extraction loss and injection deviation, as well as daily monitoring of instrument signal stability during batch detection. The detailed procedure of internal standards was as follows: 50 μL of serum sample was supplemented with 510 μL deuterated phenylalanine (240 μg/mL), 510 μL deuterated L-tryptophan (143 μg/mL) and 180 μL methanol.

**UPLC-MS analysis**

Metabolomics profiling was performed using an UPLC system (Ultimate 3000, Thermo Scientific) coupled to a Orbitrap mass spectrometer (Q Exactive Focus, Thermo Scientific). Chromatographic separation was carried out using an ACQUITY UPLC HSS T3 column (100 mm × 2.1 mm, 1.8 μm; Waters, USA) at 35 °C. The mobile phase consisted of acetonitrile (A) and 0.1% formic acid in water (B), with the following linear gradient: 0–1.0 min, 95–75% B; 1.0–2.0 min, 75–40% B; 2.0–7.5 min, 40–10% B; 7.5–10.5 min, 10–1% B; 10.5–12.5 min, 1% B; 12.5–13.0 min, 1–95% B; 13.0–15.0 min, 95% B. The flow rate was set at 0.30 mL/min with an injection volume of 3 μL, and the autosampler temperature was kept at 4 °C.

Subsequent MS detection was conducted in both positive and negative ion modes with the following parameters: heater temperature, 320 °C; sheath gas flow, 40 arb; auxiliary gas flow, 15 arb; purge gas flow, 1 arb; spray voltage, 3.5 kV (positive) and 3.2 kV (negative); capillary temperature, 350 °C; S-Lens RF level, 50%. Full-scan MS acquisition was performed over an m/z range of 80–1200 at a resolution of 70,000.

A pooled QC was run per 10 samples. All primary spectrometry files were processed in Progenesis QI (Nonlinear, Dynamics, Newcastle, UK), which performed peak picking, alignment, deconvolution and preliminary feature matching.

**Data processing and analysis**

In metabolomics analysis, MS data underwent peak detection, alignment, and correction using Waters Progenesis QI Analysis software (Nonlinear, Dynamics, Newcastle, UK). The ions with zero values present in no less than 50% were firstly excluded from the raw data. Multivariate statistical analyses, including principal component analysis (PCA) and orthogonal partial least squares discriminant analysis (OPLS-DA) were performed using SIMCA‑P software (version 14.1, Umetrics, Umea, Sweden). PCA was first applied to reveal the intrinsic metabolic variation across samples. OPLS-DA was then employed to maximize intergroup discrimination of metabolic profiles. Model quality was assessed using R2Y and Q2, with values closer to 1 indicating better stability and reliability. 200 Permutation testing was further conducted to validate model fitting; the OPLS-DA model was considered valid when all permuted Q2 and R2 values on the left were lower than the original values on the right.

Then, the ions which conform to *P* < 0.05, fold change（FC) >1.2 or FC < 0.83 were selected. These ions were deemed to have a large contribution rate to the change of the metabolic profile. Metabolite annotation follows Metabolomics Standards Initiative (MSI) guidelines (Sumner *et al.*, 2007). Identification requires consistent retention time, MS and MS/MS spectra matching authentic standards. Polar metabolites were further annotated via the library built with reference compounds and the mzCloud database (Thermo Fisher Scientific, San Jose, CA). All candidate differential features were matched to endogenous metabolites through the Human Metabolome Database (HMDB) (<http://www.hmdb.ca/>) (Wishart *et al.*, 2018), with a mass tolerance of ±5 ppm for MS1 precursor m/z matching.

Pathway enrichment analysis was conducted using the Ouyi cloud website (<https://cloud.oebiotech.com/>). Meanwhile, the hypergeometric test was used to calculate pathway enrichment *P*-values, which were adjusted via Benjamini–Hochberg multiple testing to yield false discovery rate (FDR). The formula is shown below.


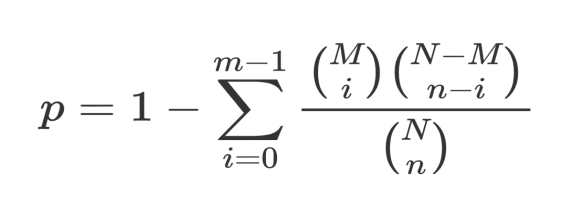


N denotes the total number of database-annotated background metabolites; n denotes the total number of database-annotated foreground metabolites; M represents the number of background metabolites assigned to a specific functional term; m represents the number of foreground metabolites annotated to the same term.

**Proteomics analysis**

**Sample preprocessing**

Five serum samples were randomly selected from both HUA patients and HCs. Briefly, 40 μL of 2.5% magnetic bead suspension was collected, washed, and resuspended in 100 μL wash buffer (0.2% trifluoroacetic acid). Next, 100 μL serum sample was mixed with the bead suspension, and the mixture was incubated at 37 °C for 1 h with gentle shaking. After magnetic separation, the supernatant was removed, and the beads were washed three times using wash buffer to capture enriched serum proteins. Subsequently, 40 μL reduction working reagent (5 mM dithiothreitol in 50 mM ammonium bicarbonate) was added to protein-bound magnetic beads. After thorough mixing, the bead mixture was incubated at 95 °C for 5 min to complete reduction. Once cooled to room temperature, 1.2 μL alkylation working solution (20 mM iodoacetamide in 50 mM ammonium bicarbonate) and 2 μL equilibration solution (50% acetonitrile) were supplemented sequentially. Finally, 5 μL trypsin digestion working solution (50 ng/μL trypsin in 50 mM ammonium bicarbonate) was added for protein digestion. The sample was mixed thoroughly and incubated at 37 °C for 2 h for enzymatic hydrolysis. The reaction was terminated, and the mixture was centrifuged at 20,000×g for 1 min. The resulting supernatant was desalted using a C18 column (15cm×75μm ID,1.6μm C18，ionopticks), followed by concentration with a vacuum centrifugal concentrator.

**LC–MS/MS high-resolution mass spectrometry detection**

Peptide concentrations were quantified using a NanoDrop system (implen N60, implen) operated with NPOS software v4.6k 16350, and absorbance was measured at 260 nm prior to separation. Nanoflow reversed-phase chromatography was carried out on a nanoElute LC platform (Bruker Daltonics) coupled to mass spectrometry. Peptides were separated in 60 min at a flow rate of 300 nL/min on a C18 column (15cm×75μm ID,1.6μm C18，ionopticks). Mobile phase A was aqueous 0.1% formic acid, while mobile phase B contained acetonitrile, water and formic acid at a volume ratio of 80:20:0.1. The elution gradient was configured as follows: 0~45 min, 22% B; 45~50 min, 22-37% B; 50~55 min, 37-80% B; 55~60 min, 80% B.

Before mass spectrometry injection, each sample was mixed according to the volume ratio of IRT: sample to be measured = 1:20 as the internal standard. Data-independent acquisition (DIA) chromatographic separation was performed on C18 column (15cm×75μm ID,1.6μm C18，ionopticks) maintained at 35 °C, with a constant flow rate of 400 nL/min. The mobile phases consisted of buffer A (0.1% formic acid in water) and buffer B (0.1% formic acid in acetonitrile). The linear elution gradient was set as follows: 0–45 min, 98–78% A; 45–50 min, 78–63% A; 50–55 min, 63–20% A; 55–60 min, 20% A held constant.

DIA mass spectrometry analysis was performed on a timsTOF Pro mass spectrometer (Bruker Daltonics), coupled with a CaptiveSpray nanoESI source. The parameters were configured as follows: capillary voltage, 1.4 kV; dry gas temperature, 180 °C; dry gas flow, 3.0 L/min; mass range, m/z 100–1700; ion mobility range, 0.7–1.3 V•s/cm²; collision energy, 20–59 eV; and TIMS ramp time, 166 ms.

**Data processing and analysis**

The mass spectrometer operated in DIA mode. Spectronaut Pulsar 18.4 (Biognosys) processed the DIA raw data using the uniprot-Homo sapiens-9606-2024.2.1.fasta database. The analysis module of the Spectronaut Pulsar software was opened, the parameter settings were set as per the software prompts (**Table S1**), and the quantitative data were exported after the analysis was completed. Differential proteins were screened based on the criteria *P* < 0.05 with FC > 1.2 or < FC < 0.83. Functional characterization of the detected proteins was achieved through Kyoto Encyclopedia of Genes and Genomes (KEGG) ([*https://www.kegg.jp/*](https://www.kegg.jp/)). STRING database was used to analyze the protein-protein interaction (PPI) networks of the differential proteins.

**Supplementary Figures**


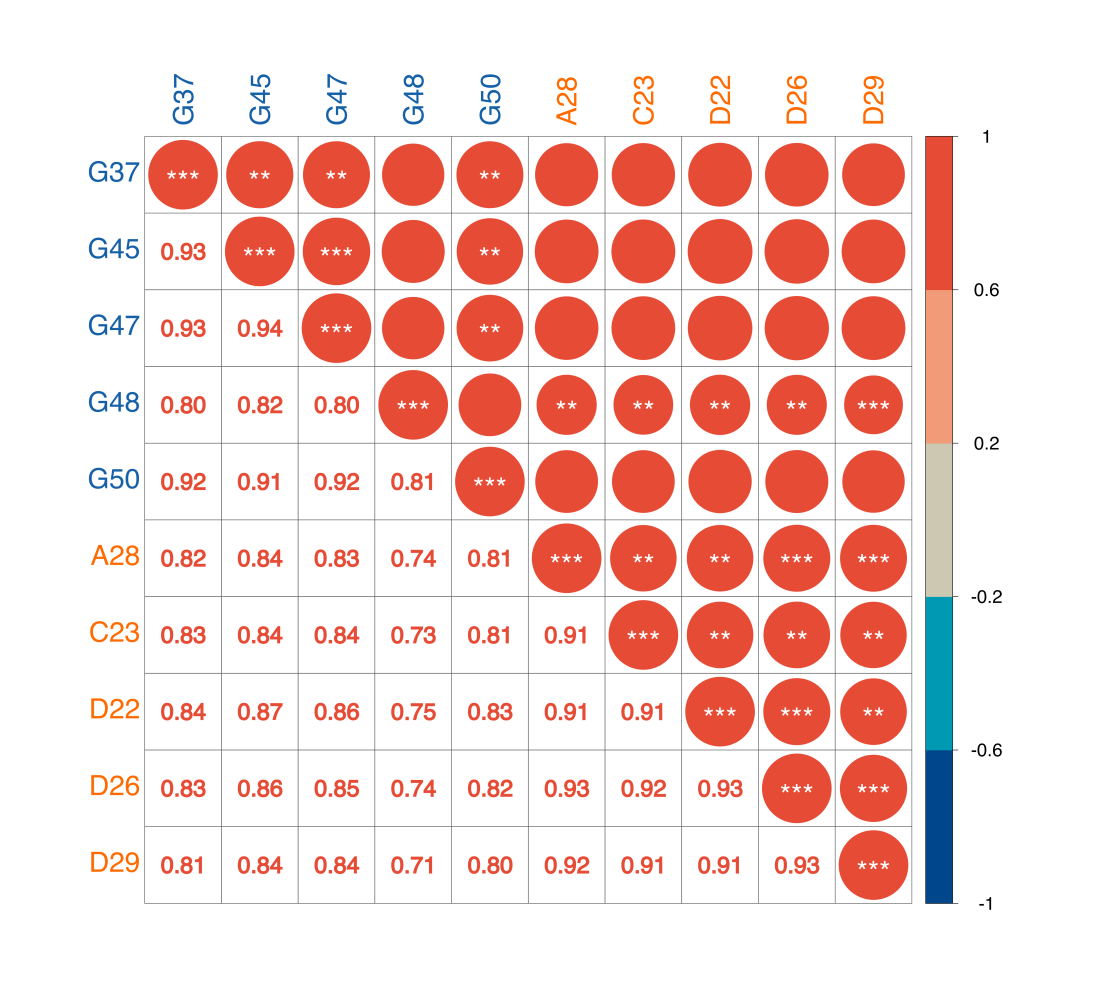


**FigS1.** Longitudinal quality control of MS. The bottom-left half of the panel represents the pairwise Pearson’s correlation coefficients of the samples, and the top-right half of the panel depicts the pairwise scatter plots from the same comparison


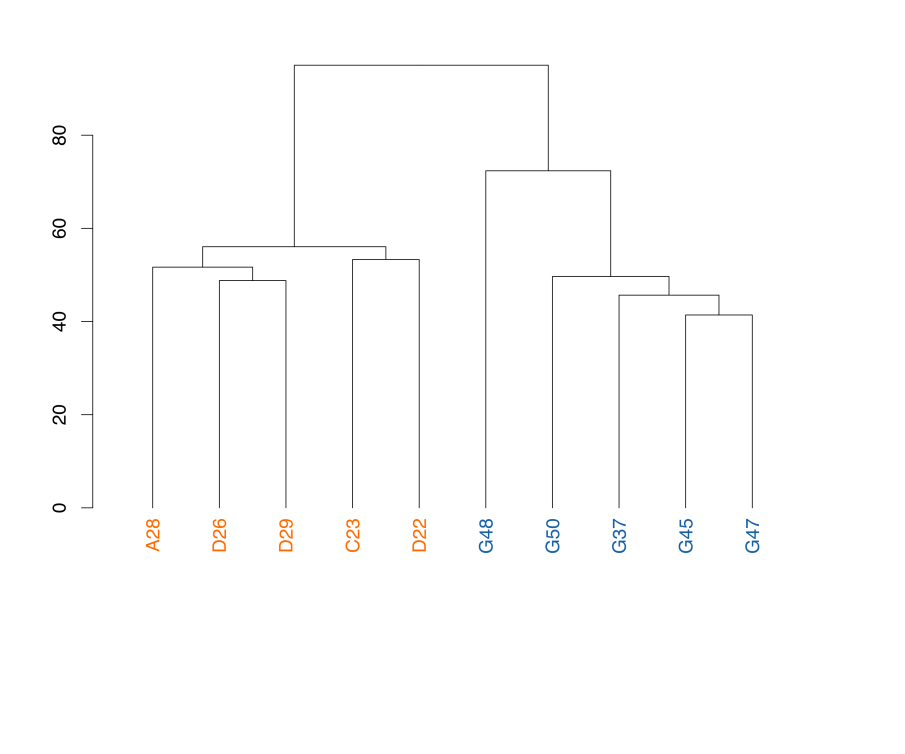


**FigS2.** Hierarchicalclustering dendrogram of sample. Each branch end represents a sample, and samples clustered within the same branch are considered to express similar or similar features, A28, D26, D29, C23 and D22 are HUA samples and G48, G50, G37, G45 and G47 are HC samples.


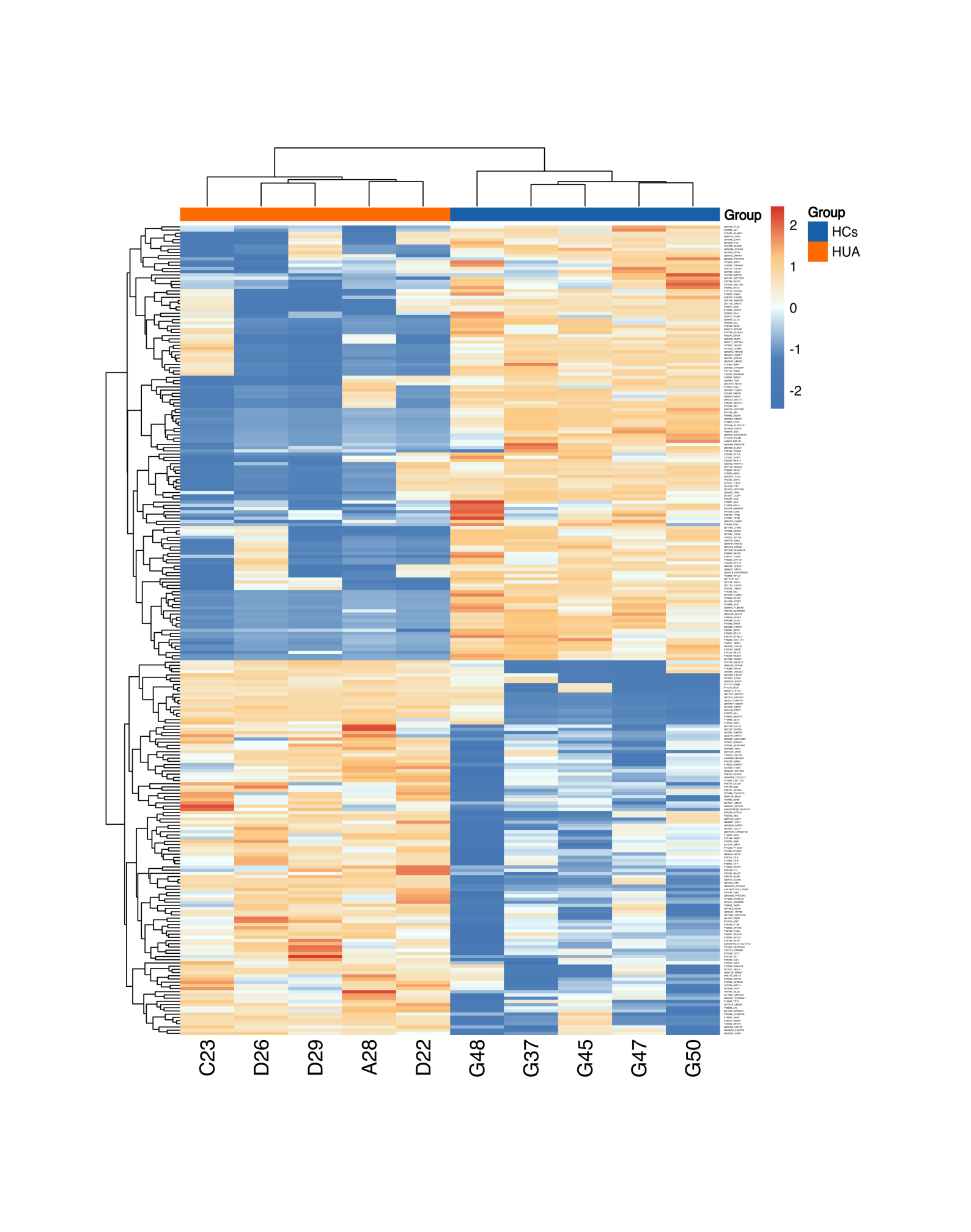


**FigS3.** Heatmap representation of abundance profile of differentially abundance proteins in HUA and HC group.


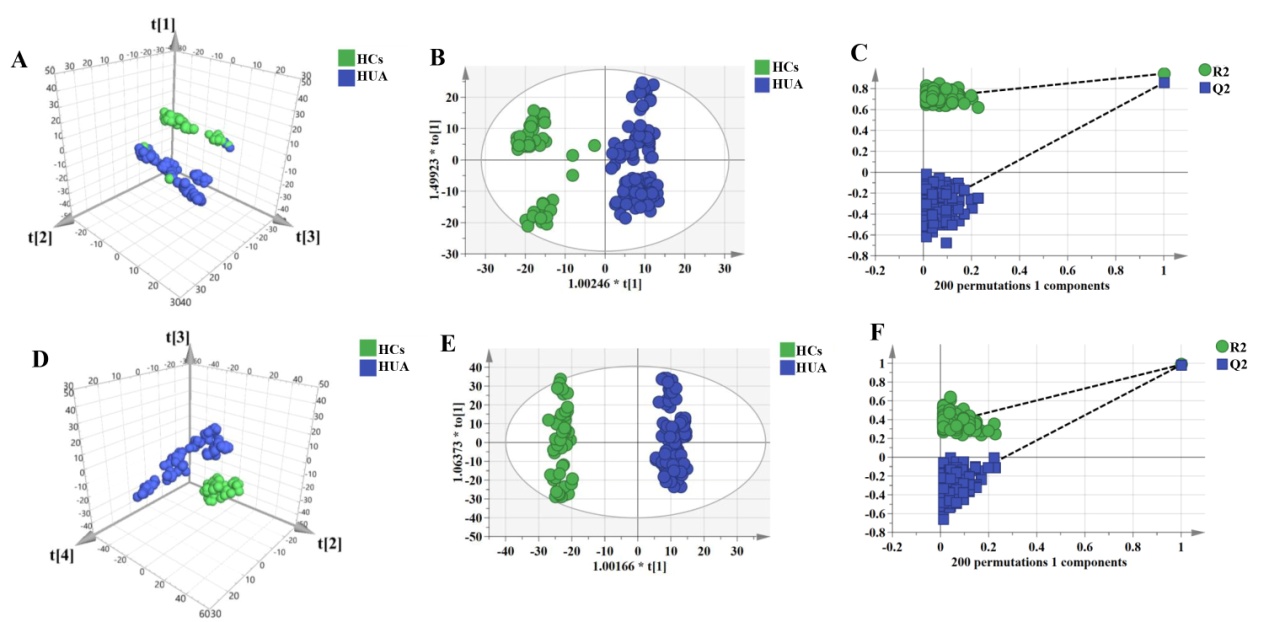


**FigS4.** Multivariate statistical analysis of the serum metabolic profiles in the validation cohort**.** (A-C) PCA, OPLS-DA scatter plots, and permutation test plots in positive ions. (D-F) PCA, OPLS-DA scatter plots, and permutation test plots in negative ions.


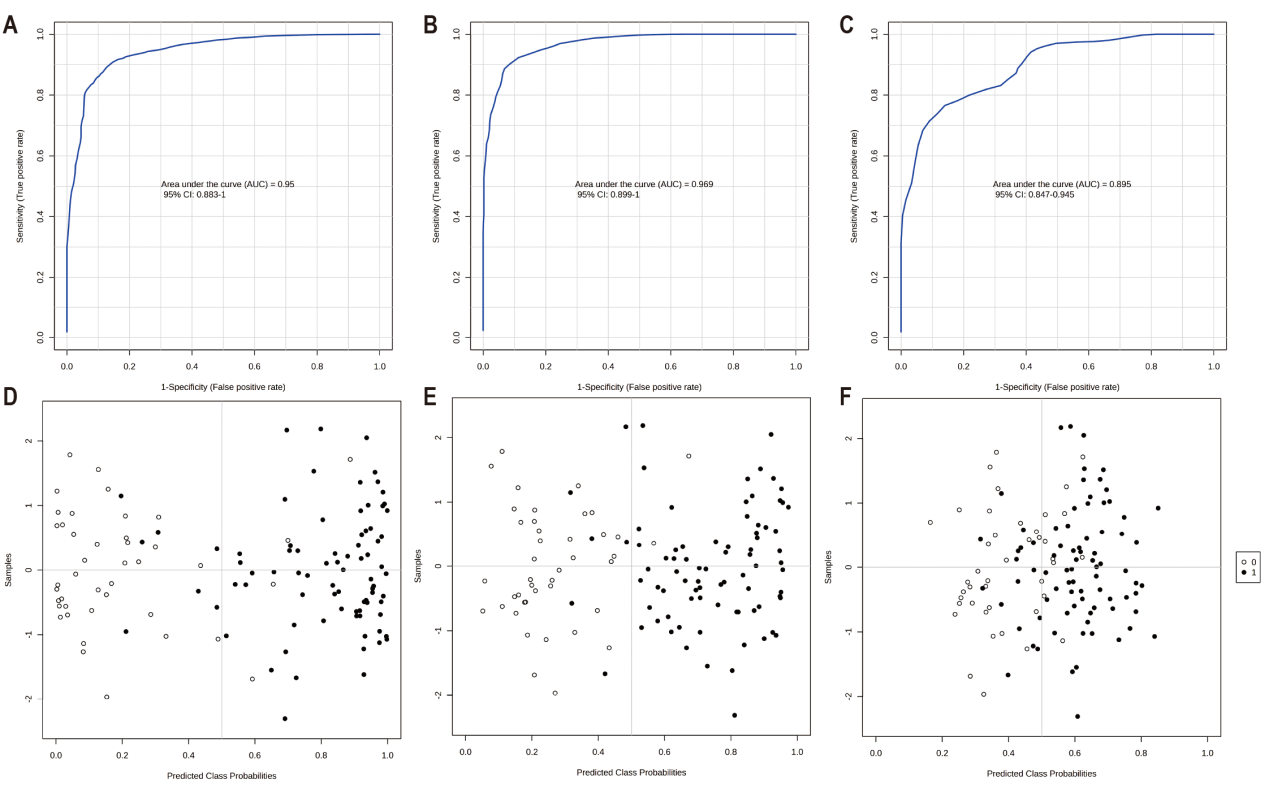


**FigS5.** Diagnosis performance of the three machine learning methods in discovery cohort. (A-C) ROC curves of SVM, RF and PLS-DA, respectively. (D-F) Confusion matrix showed SVM, RF and PLS-DA performance for classifying HUA and HC in in discovery cohort


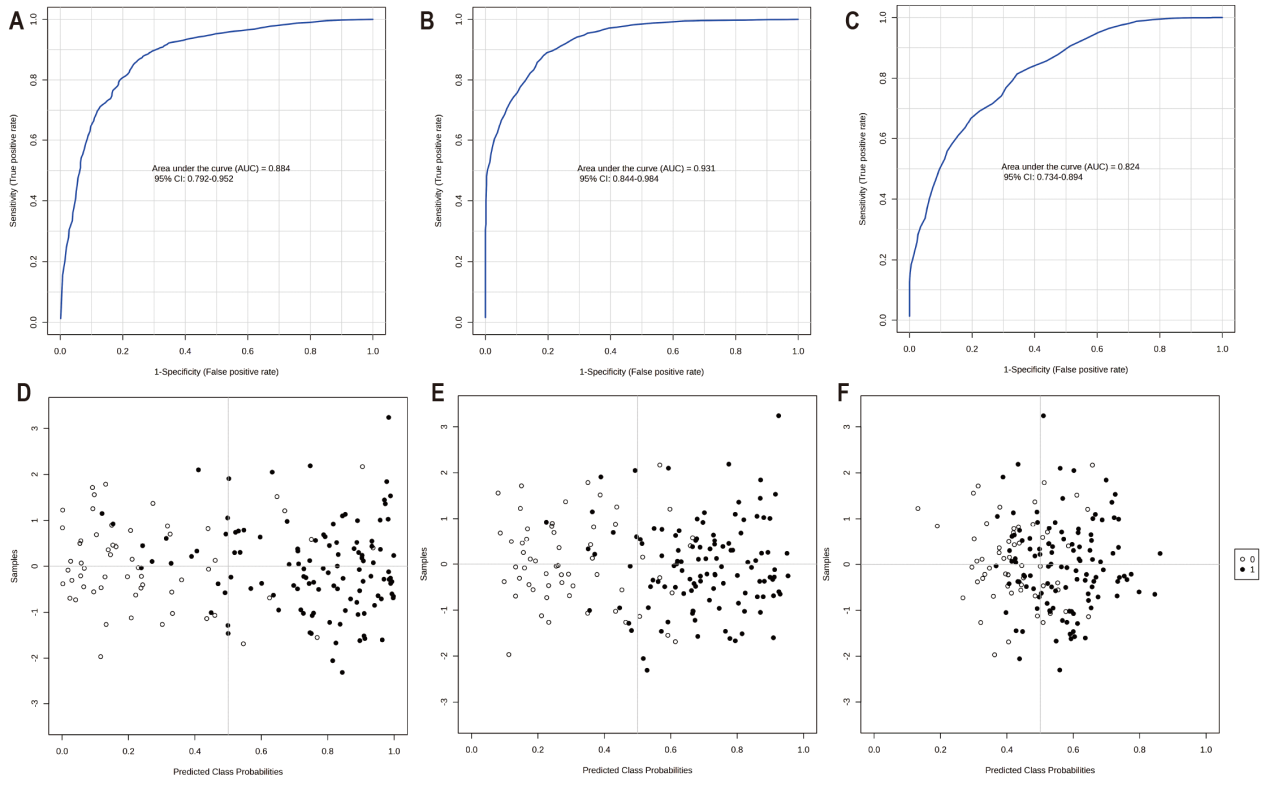


**FigS6.** Diagnosis performance of the three machine learning methods in validation cohort. (A-C) ROC curves of SVM, RF and PLS-DA, respectively. (D-F) Confusion matrix showed SVM, RF and PLS-DA performance for classifying HUA and HC in validation cohort


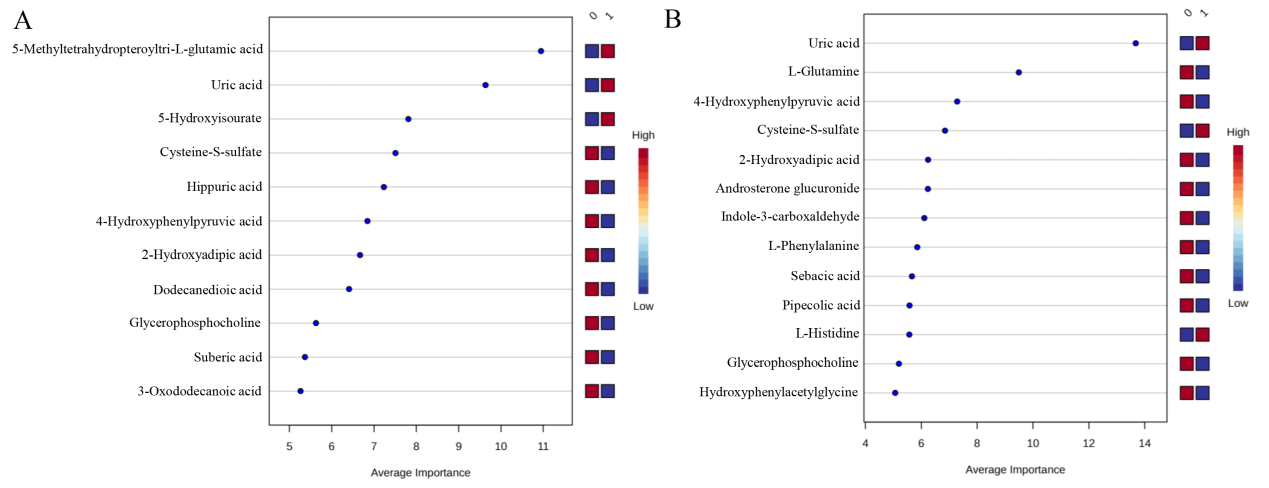


FigS7. Random forest (RF) analysis of feature importance for differential metabolites**.** (A) 11 metabolites with average importance >5 in the discovery cohort. (B) 13 metabolites with average importance in the validation cohort.


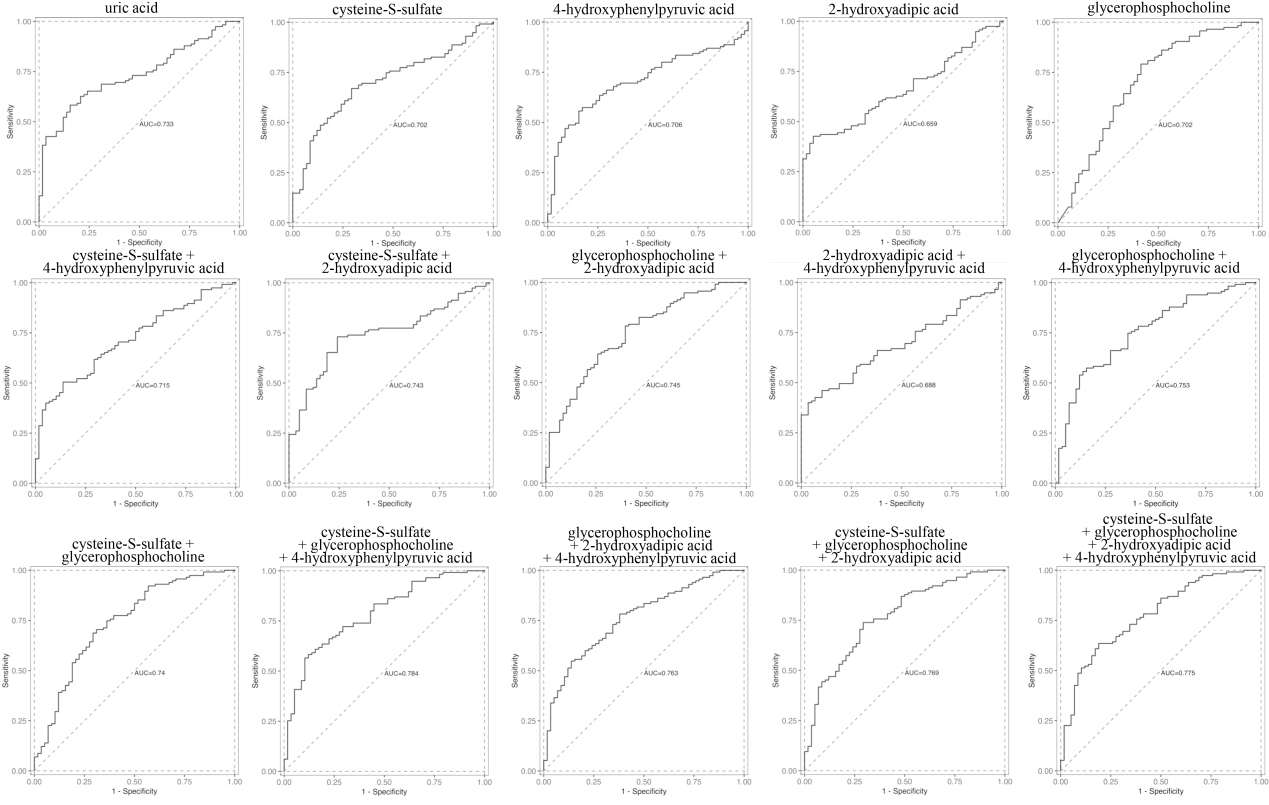


**FigS8.** Diagnosis performance of the 5 potential biomarkers and different combinations of permutations in the validation cohort between HUA patients and HC.

**Supplementary Tables**

Table S1 Key parameters for DIA data analysis

| **Items** | **Para.** |
| --- | --- |
| Missed cleavage | 2 |
| Fixed modification | Carbamidomethyl（C） |
| Variable modification | Oxidation（M）、Acetyl（Protein N-term） |
| Enzyme | Trypsin/P |
| Precursor Qvalue cutoff | 0.01 |
| Protein Qvalue cutoff | 0.01 |
| Quantity MS-Level | MS2 |

Table S2 KEGG pathway enrichment analysis of differential metabolites

| **ID** | **Term** | **p-value** | **q-value** | **Enrichment_score** |
| --- | --- | --- | --- | --- |
| hsa00591 | Linoleic acid metabolism | 7.06E-06 | 0.000769 | 9.101097 |
| hsa00360 | Phenylalanine metabolism | 0.000252 | 0.013748 | 5.386364 |
| hsa04080 | Neuroactive ligand-receptor interaction | 0.000625 | 0.022715 | 5.517738 |
| hsa04217 | Necroptosis | 0.001349 | 0.036766 | 12.56818 |
| hsa00400 | Phenylalanine, tyrosine and tryptophan biosynthesis | 0.002028 | 0.037445 | 5.386364 |
| hsa00470 | D-Amino acid metabolism | 0.002061 | 0.037445 | 3.825099 |
| hsa00350 | Tyrosine metabolism | 0.004162 | 0.058029 | 3.383741 |
| hsa00140 | Steroid hormone biosynthesis | 0.004791 | 0.058029 | 2.986499 |
| hsa04726 | Serotonergic synapse | 0.009366 | 0.096449 | 6.653743 |
| hsa04621 | NOD-like receptor signaling pathway | 0.009733 | 0.096449 | 12.56818 |
| hsa00970 | Aminoacyl-tRNA biosynthesis | 0.011411 | 0.103646 | 3.625437 |
| hsa00230 | Purine metabolism | 0.016579 | 0.132147 | 2.613186 |
| hsa00380 | Tryptophan metabolism | 0.021601 | 0.15697 | 2.72563 |
| hsa00220 | Arginine biosynthesis | 0.024399 | 0.166216 | 4.713068 |
| hsa00240 | Pyrimidine metabolism | 0.029411 | 0.187843 | 2.856405 |
| hsa04664 | Fc epsilon RI signaling pathway | 0.032743 | 0.187843 | 6.855372 |
| hsa04917 | Prolactin signaling pathway | 0.032743 | 0.187843 | 6.855372 |
| hsa04927 | Cortisol synthesis and secretion | 0.038625 | 0.196243 | 6.284091 |
| hsa00330 | Arginine and proline metabolism | 0.038678 | 0.196243 | 2.65525 |
| hsa04974 | Protein digestion and absorption | 0.040076 | 0.196243 | 3.90047 |
| hsa04934 | Cushing syndrome | 0.044874 | 0.196243 | 5.800699 |
| hsa05146 | Amoebiasis | 0.044874 | 0.196243 | 5.800699 |
| hsa00130 | Ubiquinone and other terpenoid-quinone biosynthesis | 0.04501 | 0.196243 | 2.547604 |
| hsa00410 | beta-Alanine metabolism | 0.051416 | 0.200358 | 3.534801 |
| hsa04611 | Platelet activation | 0.051468 | 0.200358 | 5.386364 |
| hsa04923 | Regulation of lipolysis in adipocytes | 0.051468 | 0.200358 | 5.386364 |
| hsa00590 | Arachidonic acid metabolism | 0.056874 | 0.212131 | 2.386364 |
| hsa03320 | PPAR signaling pathway | 0.077497 | 0.248447 | 12.56818 |
| hsa05230 | Central carbon metabolism in cancer | 0.188245 | 0.351846 | 2.513636 |
| hsa02010 | ABC transporters | 0.450828 | 0.599272 | 1.216276 |

ID: KEGG pathway ID; Term: specific name of the enriched pathway; p-value is the statistical significance p-value of pathway enrichment,; q-value is the adjusted p-value (false discovery rate, FDR); Enrichment_score is the enrichment score of the pathway.

Table S3 KEGG pathway enrichment analysis of differential proteins

| **ID** | **Term** | **p-value** | **q-value** | **Enrichment_score** |
| --- | --- | --- | --- | --- |
| hsa00730 | Thiamine metabolism | 0.010704 | 0.121908 | 12.55556 |
| hsa00030 | Pentose phosphate pathway | 0.042494 | 0.243721 | 6.075269 |
| hsa00740 | Riboflavin metabolism | 0.081897 | 0.373086 | 11.77083 |
| hsa00010 | Glycolysis / Gluconeogenesis | 0.108276 | 0.742414 | 3.560924 |
| hsa00785 | Lipoic acid metabolism | 0.145939 | 0.742414 | 6.37218 |
| hsa00340 | Histidine metabolism | 0.166976 | 0.742414 | 5.503247 |
| hsa00910 | Nitrogen metabolism | 0.174996 | 0.549402 | 5.231481 |
| hsa00020 | Citrate cycle (TCA cycle) | 0.227071 | 0.742414 | 3.90553 |
| hsa00513 | Various types of N-glycan biosynthesis | 0.294742 | 0.742414 | 2.882653 |
| hsa00500 | Starch and sucrose metabolism | 0.319648 | 0.722015 | 2.615741 |
| hsa04015 | Rap1 signaling pathway | 0.025997 | 0.198384 | 2.640187 |
| hsa04022 | cGMP-PKG signaling pathway | 0.03288 | 0.217966 | 2.802579 |
| hsa04071 | Sphingolipid signaling pathway | 0.040536 | 0.243721 | 3.087432 |
| hsa04371 | Apelin signaling pathway | 0.058865 | 0.301685 | 2.729469 |
| hsa04014 | Ras signaling pathway | 0.104309 | 0.41122 | 2.003546 |
| hsa04151 | PI3K-Akt signaling pathway | 0.164593 | 0.541298 | 1.55098 |
| hsa04010 | MAPK signaling pathway | 0.217237 | 0.620889 | 1.559051 |
| hsa04024 | cAMP signaling pathway | 0.227886 | 0.622889 | 1.637681 |
| hsa04150 | mTOR signaling pathway | 0.244156 | 0.640248 | 1.754658 |
| hsa04072 | Phospholipase D signaling pathway | 0.250683 | 0.742414 | 1.729592 |
| hsa04921 | Oxytocin signaling pathway | 0.006363 | 0.08696 | 3.598726 |
| hsa03320 | PPAR signaling pathway | 0.027307 | 0.742414 | 4.597649 |
| hsa04915 | Estrogen signaling pathway | 0.070832 | 0.344389 | 2.562358 |
| hsa04927 | Cortisol synthesis and secretion | 0.108276 | 0.742414 | 3.560924 |
| hsa04914 | Progesterone-mediated oocyte maturation | 0.116099 | 0.440746 | 2.522321 |
| hsa04924 | Renin secretion | 0.124485 | 0.742414 | 3.272201 |
| hsa04918 | Thyroid hormone synthesis | 0.138411 | 0.742414 | 3.065099 |
| hsa04614 | Renin-angiotensin system | 0.173874 | 0.742414 | 5.263975 |
| hsa04925 | Aldosterone synthesis and secretion | 0.20292 | 0.742414 | 2.397454 |
| hsa04922 | Glucagon signaling pathway | 0.230262 | 0.742414 | 2.201299 |

ID: KEGG pathway ID; Term: specific name of the enriched pathway; p-value is the statistical significance p-value of pathway enrichment,; q-value is the adjusted p-value (false discovery rate, FDR); Enrichment_score is the enrichment score of the pathway.

Table S4 Significant overlapping pathways between metabolomics and proteomics analysis

| Id | Term | p-value | q-value | Enrichment_score |
| --- | --- | --- | --- | --- |
| hsa03320 | PPAR signaling pathway | 0.00582 | 0.0529 | 16.0828 |
| hsa04923 | Regulation of lipolysis in adipocytes | 0.04584 | 0.16119 | 5.74384 |
| hsa04927 | Cortisol synthesis and secretion | 0.03433 | 0.14968 | 6.70115 |
| hsa00590 | Arachidonic acid metabolism | 0.03747 | 0.15543 | 2.68046 |
| hsa00230 | Purine metabolism | 0.01198 | 0.08702 | 2.78662 |
| hsa00240 | Pyrimidine metabolism | 0.02045 | 0.12049 | 3.14116 |
| hsa04071 | Sphingolipid signaling pathway | 0.04206 | 0.16732 | 5.36092 |

ID: KEGG pathway ID; Term: specific name of the enriched pathway; p-value is the statistical significance p-value of pathway enrichment,; q-value is the adjusted p-value (false discovery rate, FDR); Enrichment_score is the enrichment score of the pathway.

**Table S5** Candidate serum metabolites in discovery cohort and validation cohort

|  |  | **Discovery Cohort** | | | **Validation Cohort** | | |  |  |
| --- | --- | --- | --- | --- | --- | --- | --- | --- | --- |
| **NO.** | **Name** | **AUC** | **Log2 FC** | **P** | **AUC** | **Log2 FC** | **P** | **HMDB** | **KEGG** |
| 1 | 11-beta-Hydroxyandrosterone-3-glucuronide | 0.66637 | -0.36694 | 0.002920303 | 0.61589 | -0.29161 | 0.017955526 | [HMDB0010351](http://www.hmdb.ca/metabolites/HMDB0010351) | [C05643](http://www.genome.jp/dbget-bin/www_bget?C05643) |
| 2 | 13-L-Hydroperoxylinoleic acid | 0.72898 | 0.54183 | 1.11755E-05 | 0.63658 | 0.34017 | 0.008044723 | [HMDB0003871](http://www.hmdb.ca/metabolites/HMDB0003871) | [C04717](http://www.genome.jp/dbget-bin/www_bget?C04717) |
| 3 | 2-Hydroxyadipic acid | 0.71282 | 0.37837 | 1.55146E-06 | 0.65877 | 0.3058 | 0.006740368 | [HMDB0000321](http://www.hmdb.ca/metabolites/HMDB0000321) | [C02360](http://www.genome.jp/dbget-bin/www_bget?C02360) |
| 4 | 3-Oxododecanoic acid | 0.69841 | 0.78958 | 9.65545E-05 | 0.63553 | 0.4741 | 0.001716961 | [HMDB0010727](http://www.hmdb.ca/metabolites/HMDB0010727) | [C02367](http://www.genome.jp/dbget-bin/www_bget?C02367) |
| 5 | 4-Hydroxyphenylpyruvic acid | 0.74603 | 0.79929 | 1.43555E-06 | 0.70585 | 0.45739 | 0.010747208 | [HMDB0000707](http://www.hmdb.ca/metabolites/HMDB0000707) | [C01179](http://www.genome.jp/dbget-bin/www_bget?C01179) |
| 6 | 5-Hydroxyisourate | 0.75632 | -0.69205 | 1.33891E-07 | 0.64835 | -0.37294 | 0.00079526 | [HMDB0030097](http://www.hmdb.ca/metabolites/HMDB0030097) | [C11821](http://www.genome.jp/dbget-bin/www_bget?C11821) |
| 7 | 5-Methyltetrahydropteroyltri-L-glutamic acid | 0.86273 | -2.6393 | 0.000118599 | 0.69985 | -1.6139 | 9.79418E-06 | [HMDB0012177](http://www.hmdb.ca/metabolites/HMDB0012177) | [C04489](http://www.genome.jp/dbget-bin/www_bget?C04489) |
| 8 | Androsterone glucuronide | 0.70973 | -1.0277 | 0.006584401 | 0.7099 | -1.0321 | 3.85144E-05 | [HMDB0002829](http://www.hmdb.ca/metabolites/HMDB0002829) | [C11135](http://www.genome.jp/dbget-bin/www_bget?C11135) |
| 9 | Coproporphyrin III | 0.64668 | -0.68471 | 0.019712184 | 0.64903 | -0.63546 | 0.004427074 | [HMDB0000570](http://www.hmdb.ca/metabolites/HMDB0000570) | [C05770](http://www.genome.jp/dbget-bin/www_bget?C05770) |
| 10 | Cysteine-S-sulfate | 0.75015 | 1.2101 | 7.17563E-08 | 0.70225 | 0.70293 | 0.00103411 | [HMDB0000731](http://www.hmdb.ca/metabolites/HMDB0000731) | [C05824](http://www.genome.jp/dbget-bin/www_bget?C05824) |
| 11 | Dodecanedioic acid | 0.77572 | 0.91683 | 5.67858E-06 | 0.60375 | 0.4424 | 0.011408746 | [HMDB0000623](http://www.hmdb.ca/metabolites/HMDB0000623) | [C02678](http://www.genome.jp/dbget-bin/www_bget?C02678) |
| 12 | Galactosylglycerol | 0.68078 | 0.34975 | 0.002230817 | 0.63223 | 0.31108 | 0.004128122 | [HMDB0006790](http://www.hmdb.ca/metabolites/HMDB0006790) | [C05401](http://www.genome.jp/dbget-bin/www_bget?C05401) |
| 13 | Glycerophosphocholine | 0.71664 | 1.0474 | 4.82711E-06 | 0.70247 | 1.0513 | 4.99467E-07 | [HMDB0000086](http://www.hmdb.ca/metabolites/HMDB0000086) | [C00670](http://www.genome.jp/dbget-bin/www_bget?C00670) |
| 14 | Hippuric acid | 0.78601 | 0.80612 | 3.66226E-05 | 0.71394 | 0.70377 | 1.97399E-06 | [HMDB0000714](http://www.hmdb.ca/metabolites/HMDB0000714) | [C01586](http://www.genome.jp/dbget-bin/www_bget?C01586) |
| 15 | Hydroxyphenylacetylglycine | 0.68519 | -1.3005 | 0.020550308 | 0.65262 | -0.97753 | 0.022758207 | [HMDB0000735](http://www.hmdb.ca/metabolites/HMDB0000735) | [C05596](http://www.genome.jp/dbget-bin/www_bget?C05596) |
| 16 | Indole-3-carboxaldehyde | 0.63139 | 0.41535 | 0.002376483 | 0.72369 | 0.60166 | 4.15911E-07 | [HMDB0029737](http://www.hmdb.ca/metabolites/HMDB0029737) | [C08493](http://www.genome.jp/dbget-bin/www_bget?C08493) |
| 17 | Leukotriene B4 | 0.60553 | 0.3635 | 0.036543282 | 0.67121 | 0.52497 | 0.00034705 | [HMDB0001085](http://www.hmdb.ca/metabolites/HMDB0001085) | [C02165](http://www.genome.jp/dbget-bin/www_bget?C02165) |
| 18 | L-Glutamine | 0.6408 | 0.55254 | 0.000113288 | 0.70135 | 0.32604 | 0.023314384 | [HMDB0000641](http://www.hmdb.ca/metabolites/HMDB0000641) | [C00064](http://www.genome.jp/dbget-bin/www_bget?C00064) |
| 19 | L-Histidine | 0.70547 | 0.69943 | 0.000209448 | 0.62489 | 0.61162 | 0.000198289 | [HMDB0000177](http://www.hmdb.ca/metabolites/HMDB0000177) | [C00135](http://www.genome.jp/dbget-bin/www_bget?C00135) |
| 20 | L-Phenylalanine | 0.63962 | 0.49684 | 0.001358549 | 0.68651 | 0.4046 | 0.008166377 | [HMDB0000159](http://www.hmdb.ca/metabolites/HMDB0000159) | [C00079](http://www.genome.jp/dbget-bin/www_bget?C00079) |
| 21 | Oleic acid | 0.62816 | 0.34446 | 0.000976453 | 0.66372 | 0.35299 | 0.002423244 | [HMDB0000207](http://www.hmdb.ca/metabolites/HMDB0000207) | [C00712](http://www.genome.jp/dbget-bin/www_bget?C00712) |
| 22 | Phenylpyruvic acid | 0.67255 | 0.59003 | 0.001121829 | 0.65562 | 0.51363 | 0.001204099 | [HMDB0000205](http://www.hmdb.ca/metabolites/HMDB0000205) | [C00166](http://www.genome.jp/dbget-bin/www_bget?C00166) |
| 23 | Pipecolic acid | 0.61905 | 0.5981 | 0.000146688 | 0.6596 | 0.6235 | 0.002025193 | [HMDB0000070](http://www.hmdb.ca/metabolites/HMDB0000070) | [C00408](http://www.genome.jp/dbget-bin/www_bget?C00408) |
| 24 | Sebacic acid | 0.65079 | 0.56537 | 0.005145716 | 0.68456 | 0.50807 | 0.015618246 | [HMDB0000792](http://www.hmdb.ca/metabolites/HMDB0000792) | [C08277](http://www.genome.jp/dbget-bin/www_bget?C08277) |
| 25 | Suberic acid | 0.69782 | 0.49835 | 0.001550103 | 0.65952 | 0.52198 | 0.000372962 | [HMDB0000893](http://www.hmdb.ca/metabolites/HMDB0000893) | [C08278](http://www.genome.jp/dbget-bin/www_bget?C08278) |
| 26 | UA | 0.78718 | -0.30583 | 1.24617E-08 | 0.73283 | -0.33221 | 7.20407E-07 | [HMDB0000289](http://www.hmdb.ca/metabolites/HMDB0000289) | [C00366](http://www.genome.jp/dbget-bin/www_bget?C00366) |

Sumner, L.W., Amberg, A., Barrett, D., Beale, M.H., Beger, R., Daykin, C.A., Fan, T.W., Fiehn, O., Goodacre, R., Griffin, J.L., Hankemeier, T., Hardy, N., Harnly, J., Higashi, R., Kopka, J., Lane, A.N., Lindon, J.C., Marriott, P., Nicholls, A.W., Reily, M.D., Thaden, J.J. and Viant, M.R. (2007) Proposed minimum reporting standards for chemical analysis Chemical Analysis Working Group (CAWG) Metabolomics Standards Initiative (MSI). *Metabolomics*, *3*(3), 211-221. <http://doi.org/10.1007/s11306-007-0082-2>

Wishart, D.S., Feunang, Y.D., Marcu, A., Guo, A.C., Liang, K., Vázquez-Fresno, R., Sajed, T., Johnson, D., Li, C., Karu, N., Sayeeda, Z., Lo, E., Assempour, N., Berjanskii, M., Singhal, S., Arndt, D., Liang, Y., Badran, H., Grant, J., Serra-Cayuela, A., Liu, Y., Mandal, R., Neveu, V., Pon, A., Knox, C., Wilson, M., Manach, C. and Scalbert, A. (2018) HMDB 4.0: the human metabolome database for 2018. *Nucleic Acids Res*, *46*(D1), D608-d617. <http://doi.org/10.1093/nar/gkx1089>
